# DDM1 and ROS1 have a role in UV-B induced- and oxidative DNA damage in *A. thaliana*

**DOI:** 10.3389/fpls.2013.00420

**Published:** 2013-10-21

**Authors:** Julia I. Qüesta, Julieta P. Fina, Paula Casati

**Affiliations:** Centro de Estudios Fotosintéticos y Bioquímicos, Universidad Nacional de RosarioRosario, Argentina

**Keywords:** UV-B, DNA repair, chromatin remodeling, DNA methylation, arabidopsis

## Abstract

Absorption of UV-B by DNA induces the formation of covalent bonds between adjacent pyrimidines. In maize and arabidopsis, plants deficient in chromatin remodeling show increased DNA damage compared to WT plants after a UV-B treatment. However, the role of enzymes that participate in DNA methylation in DNA repair after UV-B damage was not previously investigated. In this work, we analyzed how chromatin remodeling activities that have an effect on DNA methylation affects the repair of UV-B damaged DNA using plants deficient in the expression of *DDM1* and *ROS1*. First, we analyzed their regulation by UV-B radiation in arabidopsis plants. Then, we demonstrated that *ddm1* mutants accumulated more DNA damage after UV-B exposure compared to Col0 plants. Surprisingly, *ros1* mutants show less CPDs and 6-4PPs than WT plants after the treatment under light conditions, while the repair under dark conditions is impaired. Transcripts for two photolyases are highly induced by UV-B in *ros1* mutants, suggesting that the lower accumulation of photoproducts by UV-B is due to increased photorepair in these mutants. Finally, we demonstrate that oxidative DNA damage does not occur after UV-B exposure in arabidopsis plants; however, *ros1* plants accumulate high levels of oxoproducts, while *ddm1* mutants have less oxoproducts than Col0 plants, suggesting that both ROS1 and DDM1 have a role in the repair of oxidative DNA damage. Together, our data provide evidence that both *DDM1* and *ROS1*, directly or indirectly, participate in UV-B induced- and oxidative DNA damage repair.

## Introduction

Because of their sessile condition, plants are inevitably exposed to ultraviolet-B radiation (UV-B, 290–315 nm); this energetic radiation causes direct damage to DNA, proteins, lipids, and RNA (Britt, 1996; Jansen et al., 1998; Gerhardt et al., 1999; Casati and Walbot, 2004). Absorption of UV-B by DNA induces the formation of covalent bonds between adjacent pyrimidines, giving rise to cyclobutane pyrimidine dimers (CPD) and, to a lesser extent, pyrimidine (6-4) pyrimidone photoproducts (6-4PPs) (Friedberg et al., 1995). These lesions disrupt base pairing and block DNA replication and transcription if photoproducts persist, or result in mutations if photoproducts are bypassed by error-prone DNA polymerases (Britt, 1996). Accumulation of such lesions must be prevented to maintain genome integrity, plant growth and seed viability. Thus, plants have not only developed mechanisms that filter or absorb UV-B to protect them against DNA damage (Mazza et al., 2000; Bieza and Lois, 2001), but also have different DNA repair systems to remove or tolerate DNA lesions (Hays, 2002; Bray and West, 2005; Kimura and Sakaguchi, 2006). At the genome level, the accessibility of DNA is determined by the structure of chromatin, which is subjected to epigenetic regulation. The structure of chromatin can be remodeled by three distinct processes, including covalent modifications of histones, such as phosphorylation, acetylation, methylation, ubiquitylation, sumoylation; ATP-dependent reorganization and positioning of DNA-histones; and methylation of DNA cytosine residues (Verbsky and Richards, 2001; Eberharter and Becker, 2002; Pfluger and Wagner, 2007; Vaillant and Paszkowski, 2007).

In plants, DNA methylation regulates different epigenetic phenomena, including transcriptional silencing of transposons and transgenes, defense against pathogens, regulation of imprinting as well as silencing of genes (Vongs et al., 1993; Jeddeloh et al., 1999; Bender, 2004; Chan et al., 2005; Vanyushin and Ashapkin, 2011; Yaish et al., 2011). *DECREASE IN DNA METHYLATION1* (*DDM1*), is an ATP-dependent SWI2/SNF2 chromatin remodeling factor that is required for normal patterns of genomic DNA methylation in arabidopsis (Vongs et al., 1993; Jeddeloh et al., 1999). Mutations in *DDM1* result in a rapid loss of cytosine methylation at heterochromatic repetitive sequences and a gradual depletion of methylation at euchromatic low-copy sequences over successive generations (Kakutani et al., 1996). In *ddm1* heterochromatin, DNA methylation is lost and methylation of lysine 9 is largely replaced by methylation of lysine 4 (Gendrel et al., 2002). In addition, DDM1 maintains 5S rDNA methylation patterns while silencing transcription through 5S rDNA intergenic spacers (IGS) (Kurihara et al., 2008). DDM1 also regulates gene imprinting, transposon, gene and transgene silencing, and possibly the occurrence of paramutations (Jeddeloh et al., 1998; Vielle-Calzada et al., 1999; Hirochika et al., 2000). In *ddm1* plants, there is a significant DNA decondensation at centromeric and pericentromeric regions rich in repetitive sequences and transposons; and in these mutants, some transposons become transcriptionally active or even undergo transposition (Hirochika et al., 2000; Miura et al., 2001; Singer et al., 2001; Mittelsten Scheid et al., 2002; Soppe et al., 2002; Fransz et al., 2003; Lippman et al., 2003; Probst et al., 2003; Slotkin and Martienssen, 2007; Mirouze et al., 2009; Tsukahara et al., 2009). DDM1 apparently stabilizes the activity of transposons; one of the *ddm1*-induced abnormalities was shown to be caused by insertion of CAC1, an endogenous CACTA family transposon (Miura et al., 2001). *ddm1* plants are also sensitive to NaCl stress and are deficient in DNA repair by methyl methane sulfonate (Yao et al., 2012); DDM1 participates in homologous recombination, and plants deficient in the expression of this gene show sensitivity to γ and UV-C radiation; demonstrating that DDM1 plays a role in response to DNA damage (Shaked et al., 2006).

Biochemical and genetic evidences have shown that plants possess DNA glycosylases that specifically remove 5-meC from DNA, initiating its replacement by unmethylated cytosine through a base excision repair process (Gehring et al., 2009; Roldan-Arjona and Ariza, 2009; Zhu, 2009). The *in vivo* functions of plant 5-meC DNA glycosylases are not fully understood, but they seem to contribute to the stability and flexibility of the plant epigenome. Plant 5-meC DNA glycosylases comprise a subfamily of atypical HhH-GPD enzymes, examples of enzymes in this group are the arabidopsis proteins ROS1 (repressor of silencing 1), DME (Demeter), DML2, and DML3 (Demeter-like proteins 2 and 3) (Choi et al., 2002; Gong et al., 2002; Penterman et al., 2007; Ortega-Galisteo et al., 2008). ROS1 was identified in a screen for mutants with increased silencing of the repetitive *RD29ALUC* transgene (Gong et al., 2002). Together with paralogs DML2 and DML3, ROS1 is needed to regulate the DNA methylation pathway at discrete regions across the plant genome, and probably protect the genome from excess methylation (Penterman et al., 2007; Zhu et al., 2007; Ortega-Galisteo et al., 2008). ROS1 and its homologs are bifunctional DNA glycosylases/lyases that cleave the phosphodiester backbone at the 5-meC removal site by b-elimination, generating a 3′ phospho a,b-unsaturated aldehyde at the strand break (Agius et al., 2006; Gehring et al., 2006; Morales-Ruiz et al., 2006; Penterman et al., 2007; Ortega-Galisteo et al., 2008). The final reaction product generated by ROS1 is a single-nucleotide gap flanked by 3′-phosphate and 5′-phosphate termini. The phosphate group present at the 3′ end of the single-nucleotide gap generated by ROS1 is removed by a DNA 3′phosphatase (Martínez-Macías et al., 2012). Finally, a yet unknown DNA polymerase must fill this gap with an unmethylated cytosine before a DNA ligase can seal the remaining nick. In addition to 5-meC, ROS1 also excise with less efficiency its deamination product thymine (5-methyluracil) from T_G mispairs, but do not show detectable activity on either C_G pairs or U_G mispairs; and ROS1 activity is facilitated at mismatched 5-meC residues (Morales-Ruiz et al., 2006; Ponferrada-Marín et al., 2009). The *ros1* mutation increases the telomere length in arabidopsis (Liu et al., 2010b); however, *ros1* mutants have not previously shown any differential response in DNA repair when compared to WT plants (Liu et al., 2010a).

We have previously demonstrated that arabidopsis plants deficient in 4 chromatin remodeling proteins NFC4, SDG26, HAM1, and HAM2 show more damaged DNA than WT plants after 4 h of UV-B exposure (Campi et al., 2012). In addition, plants treated with an inhibitor of histone acetyltransferases, curcumin, previous to the UV-B treatment show deficiencies in DNA repair; demonstrating that histone acetylation is important during DNA repair in arabidopsis. These results showed that chromatin remodeling, and histone acetylation in particular, are essential during DNA repair by UV-B; demonstrating that both genetic and epigenetic effects control DNA repair in plants. However, the role of enzymes that participate in DNA methylation in DNA repair after UV-B damage has not been investigated yet. Therefore, the aim of this work was to analyze the role of chromatin remodeling proteins that have a role in DNA methylation in the repair of CPDs and 6-4PPs using plants deficient in the expression of *DDM1* and *ROS1*. First, we analyzed their regulation by UV-B radiation in WT plants. Then, using plants with decreased transcript levels of *DDM1* and *ROS1*, we demonstrated that *ddm1* mutants accumulated more DNA damage after UV-B exposure compared to Col0 WT plants. Surprisingly, *ros1* mutants show less CPDs and 6-4PPs than Col0 plants after the treatment under light conditions, while the repair under dark conditions is impaired. Transcripts for two photolyases are highly induced by UV-B in *ros1* mutants, suggesting that the lower accumulation of photoproducts by UV-B is due to increased photorepair in these mutants. Finally, we here demonstrate that oxidative DNA damage does not occur after UV-B exposure in arabidopsis plants; however, *ros1* plants accumulate high levels of oxoproducts, while *ddm1* mutants have less oxoproducts than Col0 plants, suggesting that both ROS1 and DDM1 have a role in the repair of oxidative DNA damage. Together, our data provide evidence that both *DDM1* and *ROS1*, directly or indirectly, participate in UV-B induced- and oxidative DNA damage repair.

## Results

### UV-B regulation of *ddm1* and *ros1*, mutant analysis and physiological effects

Chromatin remodeling has previously been shown to be crucial for UV-B damage repair in plants (Casati et al., 2006; Campi et al., 2012). Different chromatin landscapes control the accessibility of the DNA repair machinery to damaged DNA. In several organisms, a major factor affecting chromatin accessibility is DNA methylation. Therefore, we sought to determine if enzymes that have a role in DNA methylation participate in UV-B damage repair in arabidopsis. Provided that *ros1* and *ddm1* mutants were previously reported to contain altered levels of DNA methylation in their genomes (Kakutani et al., 1996; Xia et al., 2006), they confer an adequate system to analyze how DNA methylation affects the repair of UV-B induced DNA lesions. *A. thaliana* mutants defective in *DDM1* and *ROS1* were identified in the SALK collection. For *ros1*, two independent T-DNA insertional lines, SALK_135293 and SALK_045303, with insertions in the 3′ UTR and the 16th exon, respectively, were identified by a PCR screen using gene-specific primers and one specific primer for the T-DNA left border (Figures S1, S2 and Table S1 in Supplementary Material). Insertional inactivation of *ROS1* in both lines was confirmed by RT-PCR (Figures S1, S2). For the *DDM1* gene, two independent T-DNA insertional lines, SALK_000590 and SALK_093009 (*ddm1-10*, Jordan et al., 2007), with insertions in the 16th exon and the 15th intron, respectively, were identified by a PCR screen using gene-specific primers and one specific primer for the T-DNA left border (Figure S3 and Table S1 in Supplementary Material). Decreased expression of *DDM1* in both lines was confirmed by RT-PCR (Figure S4 in Supplementary Material). *ddm1* mutants show hypomethylation in several regions of the DNA; in particular, the *AtMu1* transposon, which is usually methylated and its transposase is not transcribed in WT plants, it is actively transcribed when it is hypomethylated in *ddm1* mutants (Singer et al., 2001). Figure S5 in Supplementary Material shows that *AtMu1* is highly transcribed in the SALK_093009 mutant, while is not expressed in the Col0 plants. In addition, because DNA hypomethylation induces the misregulation of the expression of diverse genes, *ddm1* mutants show an abnormal phenotype, with small and curved leaves (Kakutani et al., 1996). The SALK_093009 mutant has already been described to show a *ddm1* mutant phenotype, showing up-regulation of genes as a consequence of hypomethylated DNA (Jordan et al., 2007). In addition, Figure S5 shows that both the SALK_093009 and the SALK_000590 mutants have a similar phenotype as that described for other *ddm1* mutants (Vongs et al., 1993; Jordan et al., 2007). The SALK_000590 mutants also show high expression of *AtMu1* and a similar phenotype as that of SALK_093009 plants (not shown), suggesting that both mutants are probably deficient in DNA methylation. It is important to mention that we have not tested the methylation profile of the SALK_093009 and the SALK_000590 mutants, but we are confident, according to the observed phenotypes, transcription activation of *AtMu1* transposon and the published data (Vongs et al., 1993; Kakutani et al., 1996; Singer et al., 2001; Jordan et al., 2007) that the two mutants behave as methylation deficient.

**Figure 1:**
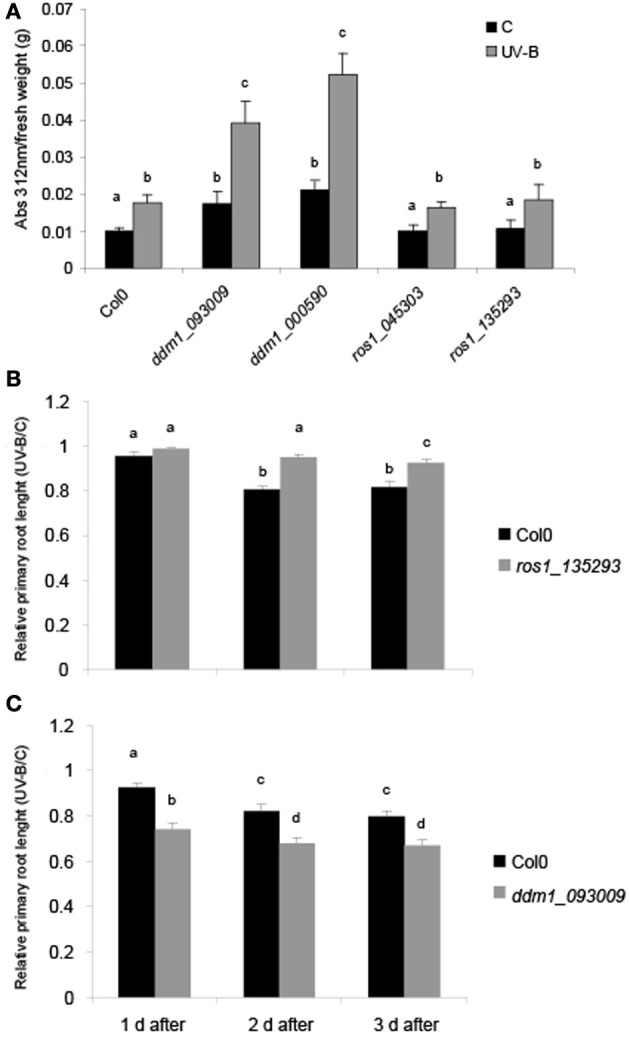
**Physiological responses in *ddm1* and *ros1* mutants after UV-B exposure. (A)** Total UV-B absorbing compounds were assayed after 4 h UV-B (UV-B) compared to untreated controls (C) in Col0 plants, and *ddm1* and *ros1* mutants. Measurements are the average of six adult leaves from six different plants. (**B** and**C**) Graph of average root lengths in Col0, *ros1* **(B)** and *ddm1* **(C)** mutants up to 3 days after a UV-B treatment. Error bars represent S.E.M. Statistical significance was analyzed using ANOVA, Tukey test with *P* < 0.05; differences from the control are marked with different letters.

We first investigated the effects of UV-B on physiological parameters in *ddm1* and *ros1* mutants. UV-B induces flavonoid accumulation such as anthocyanins and other UV sunscreens in many plants (Li et al., 1993; Landry et al., 1995; Ormrod et al., 1995). After a 4 h-UV-B treatment, the concentration of these molecules was 1.76-fold higher than under control conditions in Col0 plants. Similar increases were observed for the two *ros1* mutants analyzed (1.63- and 1.73-fold, respectively; Figure 1A). On the contrary, plants with decreased levels of *DDM1* transcript have altered accumulation of UV sunscreen photoprotectors. *ddm1* mutants showed a significantly higher increase in the level of these pigments after the UV-B treatment (2.24 and 2.16-fold increase, respectively; Figure 1A). Moreover, when pigment levels were compared in baseline control conditions in the absence of UV-B, *ddm1* mutants showed already elevated flavonoid levels similar to those in Col0 plants after the UV-B treatment. In addition, UV-B sensitivity was analyzed by inhibition of primary root elongation assays (Tong et al., 2008). One day after the end of the UV-B treatment, both Col0 and *ros1* seedlings showed a slight although significant decrease in primary root elongation compared to untreated plants (Figure 1B). However, 2 days after the treatment, *ros1* plants showed a lower decrease in primary root growth than Col0 plants. In contrast, *ddm1* seedlings showed a significant higher inhibition of root elongation by UV-B than Col0 plants (Figure 1C). Together, these results suggest that *ddm1* mutants are more sensitive to UV-B radiation than Col0 plants; while *ros1* mutants are less responsive to this radiation.

**Figure 2:**
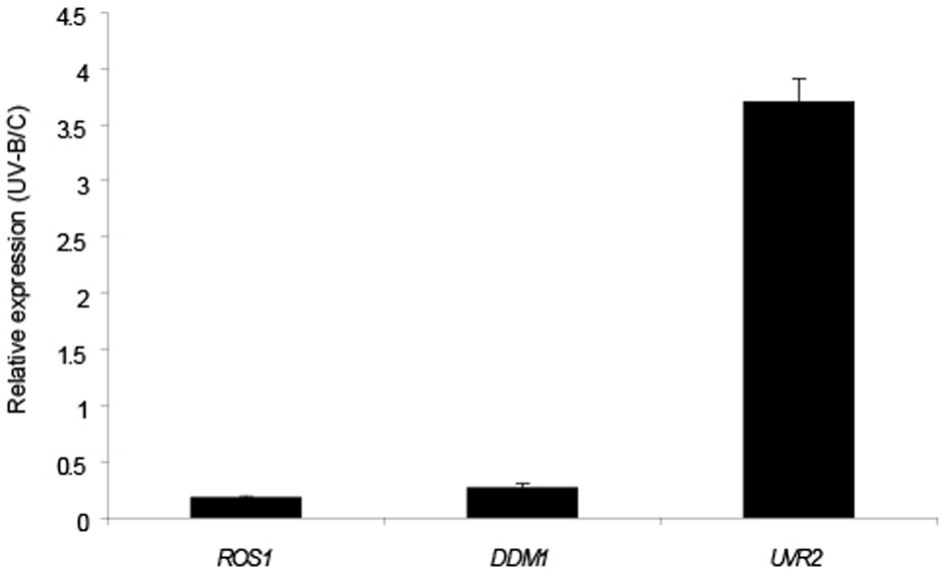
**Relative transcript levels of *A. thaliana ROS1* and *DDM1* genes measured by qRT-PCR**. Plants were irradiated with UV-B light for 4 h (UV-B) or kept under control conditions (C) as indicated in Materials and Methods. *CPK3* transcript was used for normalization and *UVR2* as a control of a UV-B inducible gene. Data show mean values ± S.E.M. of at least three independent experiments.

Previously, four arabidopsis chromatin remodeling genes *NFC4, SDG26, HAM1* and *HAM2* were reported to be induced by UV-B; and plants deficient in the expression of these genes all showed increased accumulation of CPDs compared to WT plants of the Col0 ecotype when exposed with UV-B light (Campi et al., 2012). Therefore, we investigated if *DDM1* and *ROS1* were also regulated by this radiation. 4-weeks-old Col0 (WT) plants grown in the absence of UV-B were exposed under UV-B lamps for 4 h in a growth chamber. After the treatment, leaf tissue was collected for RNA extraction and qRT-PCR analysis. Contrary to the up-regulation reported for *NFC4*, *SDG26, HAM1* and *HAM2*, Figure 2 shows that *DDM1* and *ROS1* are significantly down regulated by UV-B. The transcript of the arabidopsis CPD photolyase *UVR2* (At1g12370), a UV-B inducible gene, was used as a positive control.

### Opposing impact of ros1 and DDM1 on UV-B DNA damage repair in arabidopsis

**Figure 3:**
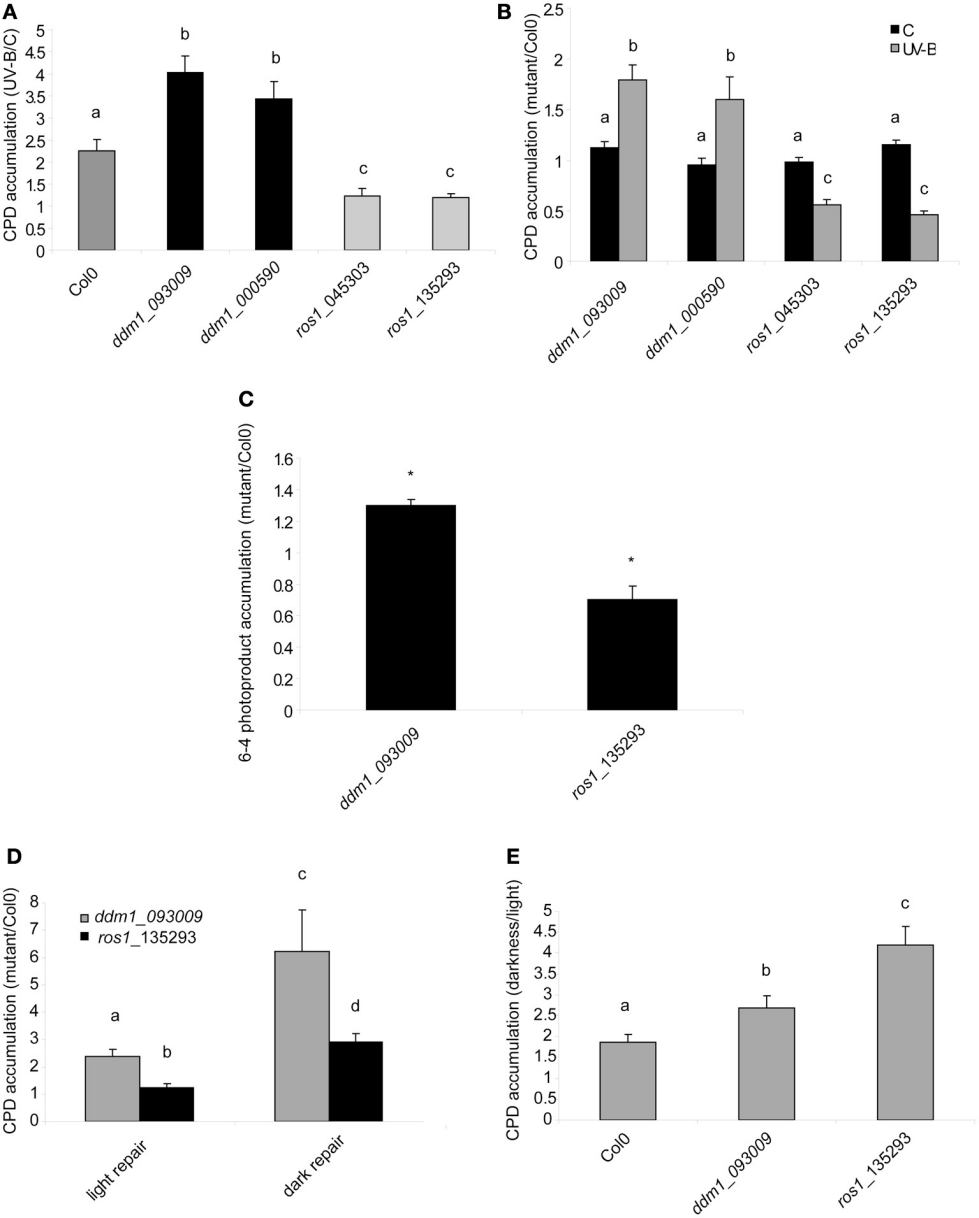
**CPD and 6-4PPs levels in the DNA of Col0, *ddm1* and *ros1* arabidopsis plants. (A)** CPD levels in DNA of UV-B treated Col0, *ddm1*, and *ros1* plants for 4 h, relative to levels under control conditions without UV-B (C). **(B)** CPD levels in DNA of *ddm1* and *ros1* plants relative to Col0 plants under control conditions without UV-B (C) and after a 4 h-UV-B treatment (UV-B). **(C)** 6-4PPs levels in DNA of *ddm1* and *ros1* plants relative to Col0 plants after a 4 h-UV-B treatment. **(D)** CPD levels in DNA of *ddm1* and *ros1* plants relative to Col0 plants after a recovery period in the absence of UV-B for 2 h. Experiments were done under conditions that allowed photorepair in the light (light) or under dark conditions (dark). **(E)** CPD levels in DNA of Col0, *ddm1* and *ros1* plants after a recovery period in the absence of UV-B for 2 h in the light relative to levels after recovery under dark conditions. Results represent the average ± S.E.M. of six independent biological replicates. Different letters denote statistical differences applying ANOVA tests using Sigma Stat 3.1. Asterisks denote statistical differences applying Student's t test (*P* < 0.05).

To test the participation of ROS1 and DDM1 in UV-B-damaged DNA repair, 4-weeks-old Col0, *ros1* and *ddm1* plants were irradiated with UV-B for 4 h. Leaf samples from control and treated plants were collected immediately after the treatment under light conditions that allow photoreactivation. Genomic DNA was extracted to evaluate CPD abundance after UV-B exposure, both in Col0 and mutant plants (Figures 3A,B). CPD levels were measured by an immunological sensitive assay; this assay detects CPDs by monoclonal antibodies specifically raised against them. 1.5 μg of DNA was used for each sample, as that there is a linear relationship of signal values of UV-B treated samples vs. the corresponding amounts of DNA loaded up to 2 μg of DNA (Lario et al., 2011). In the absence of UV-B, the steady state levels of CPDs in Col0 and mutant plants were similar [about 200 intensity of the optical density (IOD) in all samples; Figure 3B]. After 4 h UV-B exposure, unrepaired lesions accumulated in all plants (Figure 3A) CPD levels in *ddm1* mutants were significantly higher than in Col0 (Figures 3A,B). Interestingly, *ros1* mutants showed only a minor, although still significant increased accumulation of CPDs after the UV-B treatment. Consistent with the lack of UV-B sensitivity observed in the root elongation assay, *ros1* plants accumulate lower levels of CPDs than Col0 (Figures 3A,B). These results confirm the participation of ROS1 and DDM1 in UV-B damage repair and also evidence the opposing effects of these two proteins in UV-B response.

6-4PPs constitute around 25% of the DNA damage induced by UV-B radiation (Britt, 1996). We investigated how 6-4 photoproducts were accumulated in *ddm1* and *ros1* mutants. As observed for CPD accumulation, *ddm1* plants accumulated significant higher levels of 6-4PPs that Col0 plants after a 4 h-UV-B treatment, while *ros1* mutants showed lower accumulation of these products under the same conditions (Figure 3C).

### *ddm1* and *ros1* mutants have altered levels of DNA repair transcripts

**Figure 4:**
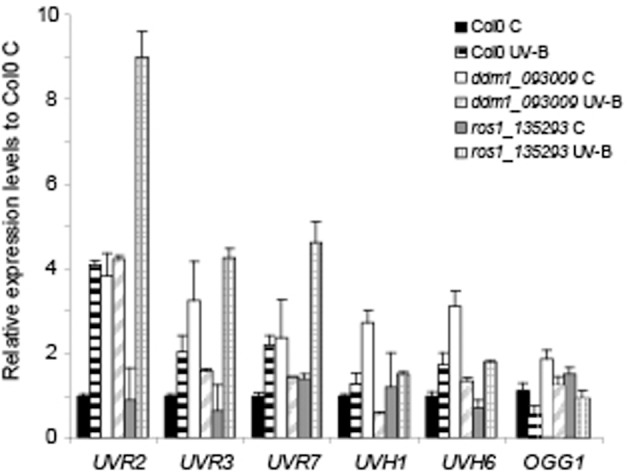
**Relative expression of DNA repair transcripts by RT-qPCR in Col0, *ddm1* (*ddm1_093009* line) and *ros1* (*ros1_135293* line)**. Levels of *UVR2, UVR3, UVR7, UVH1, UVH6*, and *OGG1* were assayed in arabidopsis plants that were irradiated with UV-B for 4 h (UV-B) or were kept under control conditions without UV-B (control, C). Expression values are relative to the values in Col0 plants under control conditions in the absence of UV-B. The *CPK3* transcript was used as a control. Data show mean values ± S.E.M. of at least three independent experiments.

The evidence of a role of DDM1 and ROS1 in UV-B damage repair prompted us to investigate their involvement in the regulation of the expression of DNA repair genes. UV-B-induced DNA damage repair is accomplished by two main pathways: nucleotide excision repair (NER) and photoreactivation (PR). Therefore, we measured the transcript levels of some selected NER and PR genes before and after UV-B exposure. First, we evaluated the expression of 2 photolyase genes: *UVR2*, encoding a CPD photolyase, and *UVR3*, encoding a 6-4 photoproduct photolyase (At3g15620). Figure 4 and Figure S6 in Supplementary Material show that both genes were up-regulated by UV-B radiation in Col0 plants after the treatment; however, *ddm1* mutants constitutively expressed high levels of both photolyases. In previous studies using different mutants that are deficient in homologous recombination and repair of damaged DNA with methylmetane sulphonate, such us *abo4* (a mutant in the DNA pol ε, Yin et al., 2009), *rfc1* (a mutant in the DNA replication factor C1; Liu et al., 2010a), and *polα* (a mutant in the DNA pol α, Liu et al., 2010b), DNA repair transcripts were highly and constitutively expressed, suggesting that in these mutants DNA repair-related genes were spontaneously induced. We hypothesize that a similar situation occurs in *ddm1* plants. In contrast, *ros1* mutants contained wild type amounts of *UVR2* and *UVR3* transcripts in the absence of UV-B and showed higher levels of both transcripts after the UV-B treatment compared to Col0 (Figure 4 and Figure S6). Thus, the lower accumulation of CPDs in *ros1* mutants after the UV-B treatment may be a result of increased photolyases activity.

On the other hand, we analyzed the expression of the NER genes *UVR7* (encoding ERCC1, a DNA excision repair protein, At3g05210), *UVH1* (encoding the RAD1 endonuclease, At5g41150), and *UVH6* (encoding a protein similar to the human helicase XPD, At1g03190). All these transcripts were induced by UV-B in the Col0 background, and this was also true for *ros1* mutants. However, after UV-B exposure the induction of *UVR7* was 3-fold higher in *ros1* plants compared to Col0 (Figure 4 and Figure S6). In *ddm1* mutants, high basal expression of these genes was detected under control conditions, as previously observed for the photolyases. Finally, we analyzed the expression of the 8-oxoguanine DNA glycosylase gene *OGG1* (At1g21710), a member of the arabidopsis base excision repair (BER) system. Although the expression of this gene was similar in Col0 and the mutants under control conditions, Col0 showed decreased levels of *OGG1* after 4 h UV-B treatment, not measured in either mutant (Figure 4).

### Lower accumulation of CPDS in *ros1* mutants are probably a consequence of increased levels of photolyases after UV-B exposure

To analyze that the decreased UV-B sensitivity of *ros1* mutants is due to increased photolyases activity, we tested the repair of CPDs in the dark and in the light after 2 h of recovery in the absence of UV-B. As expected, all plants repaired CPD damage better in the light, when photoreactivation occurs, than in the dark, when photoreactivation is absent (Figure 3E). After 2 h recovery in the light, *ros1* plants showed similar levels of CPDs as Col0 plants as a result of photoreactivation (Figure 3D). However, recovery in the dark was significantly compromised (Figures 3D,E). This result demonstrates that the low levels of CPDs accumulated in the light are probably a consequence of the higher expression of photolyases after UV-B exposure.

On the other hand, *ddm1* mutants still showed higher CPD accumulation than Col0 plants after 2 h recovery under both conditions, demonstrating that these mutants have a defect in DNA repair, probably due to a deficiency in chromatin remodeling, as already reported for other types of DNA damage (Shaked et al., 2006; Yao et al., 2012; Figure 3D). It is interesting to note that *ddm1* plants were more affected in the dark than in the light repair (Figure 3D), the reason for this may be probably because different proteins participate in the NER repair machinery (the main dark CPD repair system), while photoreactivation requires the action of only one protein, the photolyase. Therefore, chromatin remodeling activities may be more important in the dark repair, which replace the damaged DNA with new, undamaged nucleotides, to allow to spatially accomodate the different proteins that participate in this process.

Together, our results suggest that chromatin remodeling deficient *ddm1* plants have increased CPD accumulation by UV-B because DNA repair mechanisms, in particular NER proteins, may require chromatin remodeling by this enzyme for their activities. On the contrary, *ros1* mutants are also deficient in CPD dark repair, but have high photoreactivation probably as a result of increased expression of *UVR2* and *UVR3*.

### UV-B does not induce the accumulation of oxidized bases in the DNA of arabidopsis plants, but *ddm1* and *ros1* mutants are affected in oxidative damage repair

**Figure 5:**
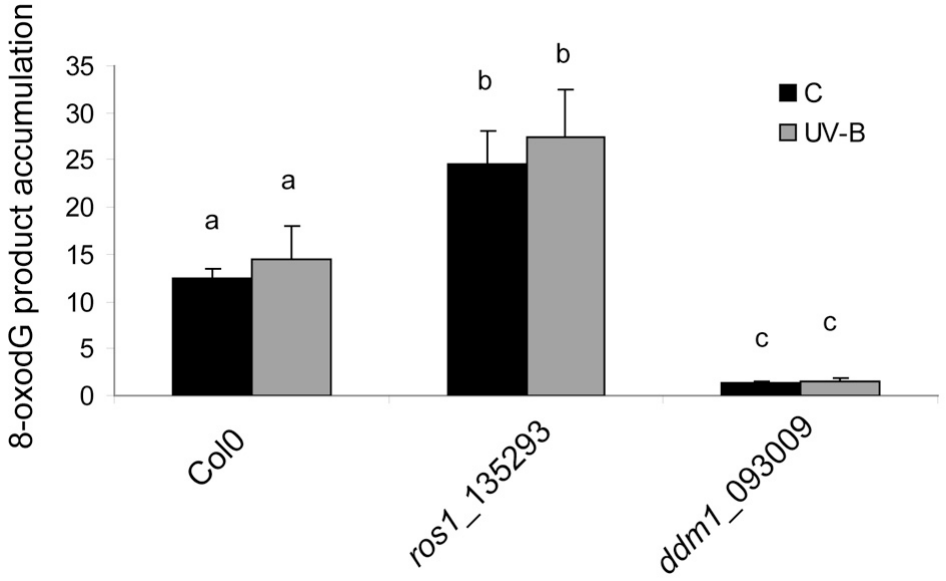
**8-oxodG levels in the DNA of Col0, *ros1* and *ddm1* arabidopsis plants**. Plants were assayed under control conditions (C) and after a 4 h UV-B treatment (UV-B). Results represent the average ± S.E.M. of six independent biological replicates. Different letters denote statistical differences applying ANOVA tests using Sigma Stat 3.1.

The results presented in Figure 4 suggest that both *ddm1* and *ros1* mutants are deficient in CPD dark repair. In plants, dark pathways fall into two major categories: NER and BER (Britt, 1996). The BER involves the removal of a single damaged base through the action of one of many lesion-specific glycosylases, which leaves the DNA sugar-phosphate backbone intact. Glycosylases and endonucleases specific for cyclobutane dimers have been observed in bacteria and bacteriophages and have been useful as diagnostic agents for the assay of UV-induced damage (Friedberg et al., 1995). On the other hand, UV-B radiation has been described to alter reactive oxygen species metabolism (Hideg et al., 2013). A wide variety of oxidative damage products are induced in DNA by hydroxyl radicals, superoxide, and nitric oxide (Britt, 1996). The most significant oxidized base is 8-hydroxyguanine (8-oxodG); thus, we investigated if UV-B produces base oxidation in arabidopsis. For this aim, we analyzed the accumulation of 8-oxodG after a 4 h UV-B treatment in Col0, *ddm1* and *ros1* mutants. Interestingly, Col0 plants did not accumulate 8-oxodG after the UV-B treatment (Figure 5). Moreover, the accumulation of 8-oxodG was neither changed in *ddm1* nor in *ros1* mutants after UV-B. However, both mutants showed significantly different accumulation of this DNA oxidation product compared to Col0 plants (Figure 5). For *ros1*, 8-oxodG accumulation was higher than Col0 plants (Figure 5). Despite that ROS1 is a glycosyltranferase of the BER repair system that has been described to remove 5-meC and T mismatched to G (Morales-Ruiz et al., 2006), its activity using oxidized bases as substrates has not been previously determined. On the other hand, *ddm1* mutants showed significantly lower levels of 8-oxodG than Col0 plants (Figure 5). This is in contrast to which was previously reported for other types of DNA damage, such as treatment with UV-C, γ-radiation and methyl methane sulfonate (Shaked et al., 2006; Yao et al., 2012), and our results in the repair of photoproducts by UV-B (Figure 3), where these mutants show higher levels of DNA damage than WT plants. In particular, *ros1* mutants show altered levels of the other 5-meC glycosylases *DML2*, *DML3* and *DME1* (Figure S7 in Supplementary Material). Therefore, it is possible that this increase in the accumulation of 8-oxodG may be due to altered expression of different glycosylases in these mutants. Together, our data provide evidence that both *DDM1* and *ROS1*, directly or indirectly, participate in oxidative DNA damage repair in arabidopsis.

## Discussion

Absorption of UV-B by DNA induces the formation of covalent bonds between adjacent pyrimidines with the formation of CPDs and 6-4PPs (Friedberg et al., 1995); overaccumulation of these lesions must be prevented to maintain genome integrity, plant growth and seed viability. Plants have evolved mechanisms that filter or absorb UV-B to protect against DNA damage (Mazza et al., 2000; Bieza and Lois, 2001), and also have DNA repair systems to remove DNA lesions (Hays, 2002; Bray and West, 2005; Kimura and Sakaguchi, 2006). The genome of plants is organized into chromatin, which limits the accessibility of DNA, affecting the rates of processes such as DNA recombination and repair. The disruption of the interactions of nucleosome–DNA or the remodeling of chromatin can stimulate or repress DNA repair. In yeast, RAD54, RAD26 and RDH54, which all belong to the switch2/sucrose non-fermenting2 (Swi2/Snf2) superfamily, participate in meiosis and also in various aspects of DNA repair, for example in homologous recombination and in nucleotide excision and transcription-coupled repair (Eisen et al., 1995; Klein, 1997; Shinohara et al., 1997). In arabidopsis, the Swi2/Snf2-related SWR1 complex, which deposits histone H2A.Z, is important for DNA repair (Rosa et al., 2013). Mutations in genes for different subunits of the SWR1 complex cause hypersensitivity to various DNA damaging agents; and even without additional genotoxic stress, these mutants show symptoms of DNA damage accumulation (Rosa et al., 2013). In maize, chromatin remodeling has been implicated in UV-B responses. Transgenic maize plants knockdown for chromatin remodeling genes were found to be acutely sensitive to UV-B at doses that do not cause visible damage to maize lacking flavonoid sunscreens (Casati et al., 2006). In maize and arabidopsis, plants deficient in chromatin remodeling show increased DNA damage compared to WT plants after a UV-B treatment (Campi et al., 2012). However, the role of enzymes that participate in DNA methylation in DNA repair after UV-B damage was not previously investigated yet. Therefore, in this work, we analyzed the role of enzymes that participate in DNA methylation in the repair of CPDs and 6-4PPs using mutant plants in *DDM1* and *ROS1*.

First, we analyzed the expression of both *DDM1* and *ROS1* by UV-B radiation in arabidopsis. Interestingly, both genes are repressed after the treatment, suggesting that DDM1 and ROS1 may have a role in UV-B responses. Therefore, their function in UV-B responses was investigated. In plants, the first line of defense when exposed to UV-B is the synthesis of protective pigments like flavonoids and UV-B absorbing pigments. In our experiments, UV-B absorbing pigments levels increased in Col0, *ddm1* and *ros1* mutants after the UV-B treatment; however, when pigment levels were compared in baseline control conditions in the absence of UV-B, *ddm1* mutants showed already elevated flavonoid levels similar to those in Col0 plants after the UV-B treatment. This demonstrates that arabidopsis plants deficient in chromatin remodeling are affected in the accumulation of UV-absorbing compounds, similarly as previously described in maize and arabidopsis chromatin remodeling deficient plants (Casati et al., 2006; Campi et al., 2012). In addition, *ddm1* seedlings showed a significantly higher inhibition of root elongation by UV-B than Col0 plants; while *ros1* roots were less affected by UV-B than those from Col0 plants. Together, these results suggest that *ddm1* mutants are more sensitive to UV-B radiation than Col0 plants; whereas *ros1* mutants are less responsive to this radiation.

In addition, we demonstrated that *ddm1* mutants accumulated more damaged DNA after UV-B exposure compared to Col0 plants. Previous studies have shown that *ddm1* plants have increased sensitivity to γ and UV-C radiation, they are susceptible to NaCl stress and are also deficient in DNA repair by methyl methane sulfonate (Shaked et al., 2006; Yao et al., 2012). Moreover, DDM1 participates in homologous recombination (Shaked et al., 2006). These data, in agreement with our results, demonstrate that DDM1 plays a role in response to DNA damage. The *ddm1* mutants used in our experiments show high expression of the *AtMu1* transposase, which is not expressed in the Col0 plants, demonstrating that these mutants have deficient methylation in some DNA regions (Singer et al., 2001). It is interesting that *ddm1* plants constitutively express high levels of DNA repair enzymes, similarly as other mutants deficient in DNA repair (Yin et al., 2009; Liu et al., 2010a,b), suggesting that in all these mutants DNA repair-related genes were spontaneously induced. However, these increased expression levels do not correlate with increased DNA repair; therefore, DDM1 may participate directly in DNA repair, and not through the regulation of the expression of DNA repair genes. A comparison of mutants in *DDM1* and *MET1*, a gene encoding a cytokine methyltransferase, suggested that DNA damage response is affected essentially by chromatin structure, while cytosine methylation was less critical (Shaked et al., 2006). Therefore, we suggest that DDM1 is important in chromatin remodeling during DNA repair of UV-B induced pyrimidine dimers.

In contrast, *ddm1* plants show significantly lower levels of 8-oxodG than Col0 plants. DDM1 has been shown to increase meiotic recombination in both male and female lineages, but the effect is not equal throughout the genome (Melamed-Bessudo and Levy, 2012). In these mutants, euchromatic regions exhibit high rates of meiotic recombination, whereas no changes are found in heterochromatic centric and pericentric areas; demonstrating the involvement of DDM1 and chromatin remodeling in genome maintenance. DDM1 regulates histone H3 and DNA methylation; upon loss of DDM1 activity, a 70% reduction in DNA methylation is induced, promoting chromatin decondensation (Jeddeloh et al., 1999; Probst et al., 2003). Therefore, the DNA demethylation *per se* or altered chromatin remodeling could make the DNA more accessible to the BER repair system, as similarly suggested for homologous recombination enzymes (Melamed-Bessudo and Levy, 2012). Interestingly, the expression levels *OGG1*, an 8-oxoguanine DNA glycosylase of the BER, is similar in *ddm1* and *Col0* plants, so increased repair of 8-oxodG cannot be explained by changes in the activity of this enzyme. However, we cannot rule out that other glycosylases or repair enzymes may be up-regulated in the *ddm1* mutants, for example by activation of silent genes from hypomethylated chromosomes.

On the other hand, in our experiments, *ros1* showed less CPDs and 6-4PPs than Col0 plants after a UV-B treatment under light conditions; however, CPD accumulation after a 2 h recovery in the dark was higher in the mutants than in Col0. The results presented here show that transcripts for two photolyases, *UVR2* (a CPD photolyase) and *UVR3* (a 6-4PPs photolyase) are highly induced by UV-B in *ros1*, suggesting that the lower accumulation of photoproducts by UV-B may be due to increased photorepair in these mutants. This higher photorepair correlates with lower inhibition of primary root elongation by UV-B, suggesting that these mutants have higher UV-B tolerance than WT plants. On the contrary, *ros1* plants accumulate elevated levels of 8-oxodG in the DNA; therefore, ROS1 may have a role in the repair of oxidative DNA damage. Interestingly, ROS1 is a DNA glycosylase that has been described to remove 5-meC and T mismatched to G (Morales-Ruiz et al., 2006), but its activity using oxidized bases as substrates has not been previously determined. Several *ros1* suppressors have been identified, including replication protein A2 (RPA2A/ROR1) (Xia et al., 2006), DNA polymerase α (Liu et al., 2010b), DNA polymerase ε (Yin et al., 2009) and TOUSLED (Wang et al., 2007). These mutants release the TGS of 35S-NPTII and increase the expression of transcriptionally active information, but they do not change the DNA methylation state when mutated. All *ros1* suppressors described above are sensitive to DNA damage, they respond to the damage with constitutive expression of DNA damage related genes, and most of them also have a high homologous recombination rate (Xia et al., 2006; Wang et al., 2007; Yin et al., 2009), suggesting that the silencing of chromatin is closely related with DNA replication, DNA repair and homologous recombination (Probst et al., 2009). However, with the exception that *ros1* mutation increases the telomere length in arabidopsis (Liu et al., 2010b), *ros1* mutants have not previously shown any differential response in DNA repair when compared to WT plants (Liu et al., 2010a). Our results suggest that *in vivo*, ROS1 may also have a role in the repair of 8-oxodG. Alternatively, a mutation in ROS1 may affect the expression of other glycosylases specific for 8-oxodG, similarly as determined for the *UVR2* and *UVR3* photolyases in this work. *ros1* plants show altered levels of the other 5-meC glycosylases *DML2, DML3* and *DME1*; thus, it is possible that this increase in the accumulation of 8-oxodG may be due to altered expression levels of different glycosylases in these mutants.

We have previously demonstrated that chromatin remodeling is essential during DNA repair by UV-B (Campi et al., 2012). In particular, because histone H3 and H4 acetylation is increased by UV-B (Casati et al., 2008), the effect of histone acetylation on DNA repair was previously analyzed, and our results demonstrated that when plants are pre-treated with curcumin, a histone acetylase inhibitor, DNA repair was impaired (Campi et al., 2012). Interestingly, in *sdg26* mutants (*SDG26* encodes a histone methyltransferase), a curcumin treatment previous to UV-B irradiation induced a significantly higher accumulation of CPDs than curcumin-treated WT plants. Therefore, a deficiency in the expression of a histone methyltransferase interferes directly or indirectly with the DNA damage repair mediated by histone acetylation, suggesting that both processes, histone acetylation and methylation, act synergistically during UV-B induced damage repair. In this manuscript, we show that enzymes that participate in DNA methylation are also important during DNA repair by UV-B, demonstrating that both genetic and epigenetic effects control DNA repair in plants.

Together, the results presented here demonstrate the participation of DDM1 and ROS1 in DNA repair after UV-B damage and oxidation. We propose that, in *ddm1* mutants, DNA demethylation *per se* or altered chromatin remodeling could affect accessibility to DNA repair systems. On the contrary, we suggest that in *ros1* mutants, lower accumulation of photoproducts is due to increased levels of photolyases by UV-B. Finally, ROS1, besides its demonstrated role as a 5-meC glycosylase, it could also act as an oxoproduct glycosidase.

## Materials and methods

### Plant material, growth conditions and irradiation protocols

The *A. thaliana* ecotype Columbia (Col0) was used for all the experiments. The T-DNA insertion mutants were obtained from the SALK T-DNA insertion mutant collection (Alonso et al., 2003). Mutants lines used are shown in Figures S1–S4 in Supplemental data. Arabidopsis plants were sown directly on soil and placed at 4°C in the dark. After 3 days, pots were transferred to a greenhouse and plants were grown at 22°C under a 16 h/8 h light/dark regime. Plants were exposed 4 h to UV-B radiation (315 nm) from UV-B bulbs (2 W m^−2^ UV-B and 0.65 W m^−2^ UV-A, Bio-Rad, Hercules, California) in a growth chamber, both in the presence or the absence of white light, and control plants were treated with the same plants covered with a polyester film (0.04 W m^−2^ UV-B, 0.4 W m^−2^ UV-A). Adult leaf samples from 4-weeks-old plants were collected immediately after irradiation, or 2 h after the end of the UV-B treatment, both under light and under dark conditions.

### Identification of insertional T-DNA mutants

The genotype of plants with T-DNA constructs was determined using a PCR-based approach. Initial screening was performed using genomic DNA isolated from leaves by a modified cetyl-trimetyl-ammonium bromide (CTAB) method (Sambrook and Russel, 2001) and three combinations of primers. Two primers hybridize to specific genomic sequences (Table S1) and one primer is located inside the left border of the T-DNA. The presence or absence of the T-DNA insertion in the genes allowed the identification of homozygous, heterozygous and WT plants.

RT-PCR for expression analyses in the knockout T-DNA lines were carried out in a 25 μ l final volume containing 1X buffer Taq DNA polymerase, 3 mM MgCl_2_, 0.2 mM dNTP, 0.25 μ M of each primer, 0.625 U Taq DNA polymerase (Invitrogen, Carlsbad, California). Cycling were performed under the following conditions: 2 min denaturation at 95°C, 35 cycles of 10 s denaturation at 95°C, 15 s annealing at 57°C, 30 s amplification at 72°C and a final 7 min amplification at 72°C. RT-PCR products were separated on a 1% (w/v) agarose gel and stained with SYBR Safe (Invitrogen).

### Quantitative RT-PCR

Total RNA was isolated from about 100 mg of tissue using the TRIzol reagent (Invitrogen) as described by the Manufacture's Protocol. The RNA was incubated with RNase-free DNase I (1 U/ml) following the protocol provided by the manufacturer to remove possible genomic DNA. Then, RNA was reverse-transcribed into first-strand cDNA using SuperScript II reverse transcriptase (Invitrogen) and oligo-dT as a primer. The resultant cDNA was used as a template for qPCR amplification in a MiniOPTICON2 apparatus (Bio-Rad), using the intercalation dye SYBRGreen I (Invitrogen) as a fluorescent reporter and Platinum Taq Polymerase (Invitrogen). Primers for each of the genes under study were designed using the PRIMER3 software (Rozen and Skaletsky, 2000) in order to amplify unique 150–250 bp products (Table S2 in Supplementary Material). Amplification conditions were carried out under the following conditions: 2 min denaturation at 94°C; 40 cycles at 94°C for 10 s, 57°C for 15 s, and 72°C for 30 s, followed by 10 min extension at 72°C. Three replicates were performed for each sample. Melting curves for each PCR were determined by measuring the decrease of fluorescence with increasing temperature (from 65 to 98°C). PCR products were run on a 2% (w/v) agarose gel to confirm the size of the amplification products and to verify the presence of a unique PCR product. Gene expressions were normalized to the *A. thaliana* calcium dependent protein kinase3 (*CPK3*, Table S2). The expression of this gene has been previously reported to remain unchanged by UV-B (Ulm et al., 2004).

### DNA damage analysis

The induction of CPD, 6-4 photoproducts and 8-oxodG was determined using an assay described in detail previously (Stapleton et al., 1993), using monoclonal antibodies specific to CPDs (TDM-2), 6-4 photoproducts (64M-2) and 8-oxodG (N45.1 obtained from Cosmo Bio Co., Ltd., Japan). After the treatments, plant samples (0.1 g) were collected and immediately immersed in liquid nitrogen and stored at −80°C. The 1.5 μg (for CPD assays), 20 μ g (for 6-4 photoproduct assays) and 2 μg (for 8-oxodG assays) of the extracted DNA by a modified cetyl-trimetyl-ammonium bromide (CTAB) method was denatured in 0.3 M NaOH for 10 min and sextuplicate biological replicates were dot blotted onto a nylon membrane (Perkin Elmer Life Sciences, Waltham, Massachusetts). The membrane was incubated for 2 h at 80°C and then it was blocked in TBS (20 mM Tris-HCl, pH 7.6,137 mM NaCl) containing 5% dried milk for 1 h at room temperature or overnight at 4°C. The blot was then washed with TBS and incubated with the different antibodies (1:2000 in TBS) overnight at 4°C with agitation. Unbound antibody was washed away and secondary antibody (BioRad) conjugated to alkaline phosphatase (1:3000) was added. The blot was then washed several times followed by the addition of the detection reagents NBT and BCIP. Quantification was achieved by densitometry of the dot blot using ImageQuant software version 5.2. DNA concentration was fluormetrically determined using the Qubit dsDNA assay kit (Invitrogen), and checked in a 1% (w/v) agarose gels after quantification. DNA concentration was determined spectrophotometrically at 260 and 280 nm in the microplate reader (Biotek XS Power Wave) using the KC Junior computer program, and checked in a 1% (w/v) agarose gel after quantification.

### Root length measurements

Petri dish-grown seedlings, surface-sterilized seeds were grown on MS growth medium and were held vertical in a growth chamber. Then, seedlings were UV-B treated for 2 h and kept in the absence of UV-B for 3 days. Plates were photographed before the treatment, and 24, 48, and 72 h after the end of the treatment, and the images were analyzed using the ImageJ program. Root lengths were determined by measuring the length of a line traced along the root.

### Pigment measurements

UV-absorbing pigments (absorbance at 312 nm) were determined as described in Casati and Walbot (2004).

### Statistical analysis

Statistical analysis was done using ANOVA models (Tukey test) using untransformed data with Sigma Stat 3.1.

## Author contributions

Julia I. Qüesta, Julieta Fina and Paula Casati designed the experiments and analyzed the data. Julia I. Qüesta and Julieta Fina did the experiments. Paula Casati wrote the article. Julia I. Qüesta, Julieta Fina and Paula Casati edited the manuscript.

### Conflict of interest statement

The authors declare that the research was conducted in the absence of any commercial or financial relationships that could be construed as a potential conflict of interest.

## Supplementary material

Figure S1 **(A)** Location of the T-DNA insertion in the *ROS1* gene (SALK_135293 line). Exons are represented by blue boxes, introns by thin black lines and the UTR regions by light gray boxes. The T-DNA insertion is indicated as a triangle. **(B)** Analysis of the PCR products separated in 1% (w/v) agarose gels. The PCR reactions were done using genomic DNA from Col0 and SALK_135293 plants. Lanes 1 show the PCR products obtained for a WT plant using the SALK_135293 F and SALK_135293 R primers; while lanes 2 show the PCR products obtained for homozygous mutant plants using the Lb and SALK_13293 R primers. **(C)** Transcript levels were evaluated by RT-PCR followed by agarose gels on cDNAs obtained from RNA extracted from the mutant or WT lines. Amplifications were performed using ROS1 F and ROS1 R primers, which are specific for the *ROS1* transcript. As a control, primers for the *RPL10B* transcript were used.

Figure S2 **(A)** Location of the T-DNA insertion in the *ROS1* gene (SALK_045303 line). Exons are represented by blue boxes, introns by thin black lines and the UTR regions by light gray boxes. The T-DNA insertion is indicated as a triangle. **(B)** Analysis of the PCR products separated in 1% (w/v) agarose gels. The PCR reactions were done using genomic DNA from Col0 and SALK_045303 plants. Lanes 1 show the PCR products obtained for a WT plant using the SALK_045303 F and SALK_045303 R primers; while lanes 2 show the PCR products obtained for homozygous mutant plants using the Lb and SALK_045303 F primers. **(C)** Transcript levels were evaluated by RT-PCR followed by agarose gels on cDNAs obtained from RNA extracted from the mutant or WT lines. Amplifications were performed using ROS1 F and ROS1 R primers, which are specific for the *ROS1* transcript. As a control, primers for the *RPL10B* transcript were used.

Figure S3 **(A, C)** Location of the T-DNA insertion in the *DDM1* gene (SALK_000590 **(A)** and SALK_093009 **(C)** lines). Exons are represented by blue boxes, introns by thin black lines and the UTR regions by light gray boxes. The T-DNA insertion is indicated as a triangle. **(B)** Analysis of the PCR products separated in 1% (w/v) agarose gels. The PCR reactions were done using genomic DNA from Col0 and SALK_000590 plants. Lanes 1 show the PCR products obtained for a WT plant using the SALK_000590 F and SALK_000590 R primers; while lanes 2 show the PCR products obtained for homozygous mutant plants using the Lb and SALK_000590 F primers. **(D)** Analysis of the PCR products separated in 1% (w/v) agarose gels. The PCR reactions were done using genomic DNA from Col0 and SALK_093009 plants. Lanes 1 show the PCR products obtained for a WT plant using the SALK_093009 F and SALK_093009 R primers; while lanes 2 show the PCR products obtained for homozygous mutant plants using the Lb and SALK_093009 R primers.

Figure S4 **Expression of *DDM1* transcripts in Col0 plants and *ddm1* mutants**. Transcript levels were evaluated by RT-PCR followed by agarose gels **(A)** and RT-qPCR **(B)** on cDNAs obtained from RNA extracted from the mutant or WT lines. Amplifications were performed using DDM1 F and DDM1 R primers, which are specific for the *DDM1* transcript. As controls, primers for the *RPL10B* **(A)** and *CPK3* **(B)** transcripts were used. **(B)** Expression values are relative to the values in Col0 plants. Data show mean values ± S.E.M. of at least three independent experiments.

Figure S5 **Phenotypes of *ddm1* mutants. (A)** Images of 4-weeks-old Col0 and SALK_093009 plants. **(B)** Images of 6-weeks-old Col0 and SALK_093009 plants. **(C)** Analysis of *AtMu1* transposase expression in Col0 and SALK_093009 plants. Transcript levels were evaluated by RT-PCR followed by agarose gels on cDNAs obtained from RNA extracted from the mutant or WT lines.

Figure S6 **Relative expression of DNA repair transcripts by RT-qPCR in Col0, *ddm1* (*ddm1_000509* line) and *ros1* (*ros1_045303* line)**. Levels of *UVR2*, *UVR3*, *UVR7*, *UVH1*, *UVH6*, and *OGG1* were assayed in arabidopsis plants that were irradiated with UV-B for 4 h (UV-B) or were kept under control conditions without UV-B (control). Expression values are relative to the values in Col0 plants under control conditions in the absence of UV-B. The *CPK3* transcript was used as a control. Data show mean values ± S.E.M. of at least three independent experiments.

Figure S7 **Relative expression of DNA glycosidase transcripts by RT-qPCR in *ros1* plants**. Levels of *DML2*, *DML3* and *DME1* were assayed in arabidopsis plants that were irradiated with UV-B for 4 h (UV-B) or were kept under control conditions without UV-B (control). Expression values are relative to the values in Col0 plants under control conditions in the absence of UV-B. The *CPK3* transcript was used as a control. Data show mean values ± S.E.M. of at least three independent experiments.

Table S1 **Primers used for identification of homozygous mutant lines**.

Table S2 **Primers used for RT-qPCR**.
