# Supplementary material for: DDM1 and ROS1 have a role in UV-B induced- and oxidative DNA damage in A. thaliana
Source: Front Plant Sci. 2013 Oct 21;4:420. doi: 10.3389/fpls.2013.00420 (PMC3801088; doi:10.3389/fpls.2013.00420)
Supplement: Figure S1 — (A) Location of the T-DNA insertion in the ROS1 gene (SALK_135293 line). Exons are represented by blue boxes, introns by thin black lines and the UTR regions by light gray boxes. The T-DNA insertion is indicated as a triangle. (B) Analysis of the PCR products separated in 1% (w/v) agarose gels. The PCR reactions were done using genomic DNA from Col0 and SALK_135293 plants. Lanes 1 show the PCR products obtained for a WT plant using the SALK_135293 F and SALK_135293 R primers; while lanes 2 show the PCR products obtained for homozygous mutant plants using the Lb and SALK_13293 R primers. (C) Transcript levels were evaluated by RT-PCR followed by agarose gels on cDNAs obtained from RNA extracted from the mutant or WT lines. Amplifications were performed using ROS1 F and ROS1 R primers, which are specific for the ROS1 transcript. As a control, primers for the RPL10B transcript were used. [file Presentation1.PDF]

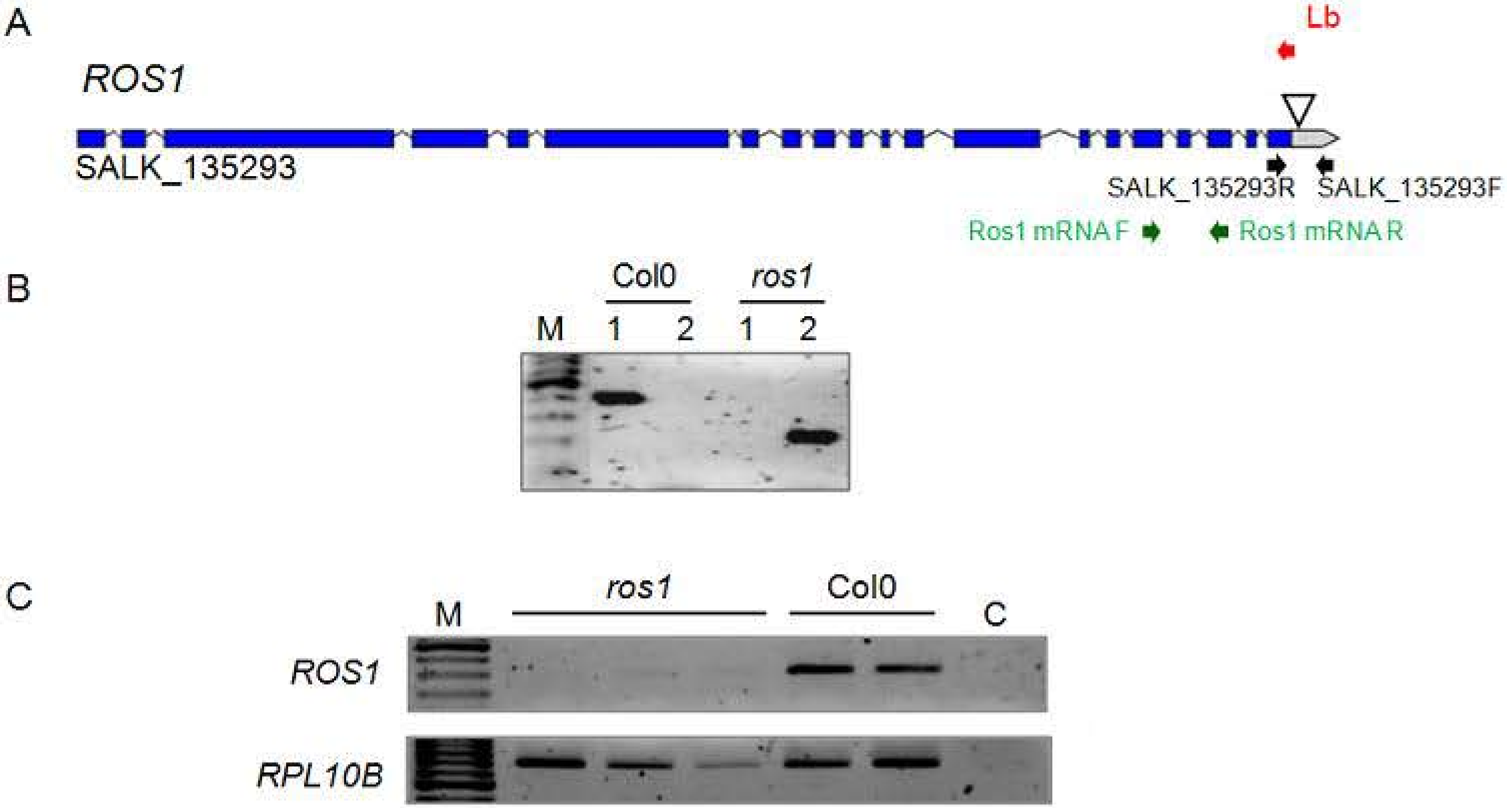

A
Lb
ROS1
SALK_135293
SALK_135293R
SALK_135293F
Ros1 mRNA F
Ros1 mRNA R
B
Col0
ros1
M
1
2
1
2
C
M
ros1
Col0
C
ROS1
RPL10B

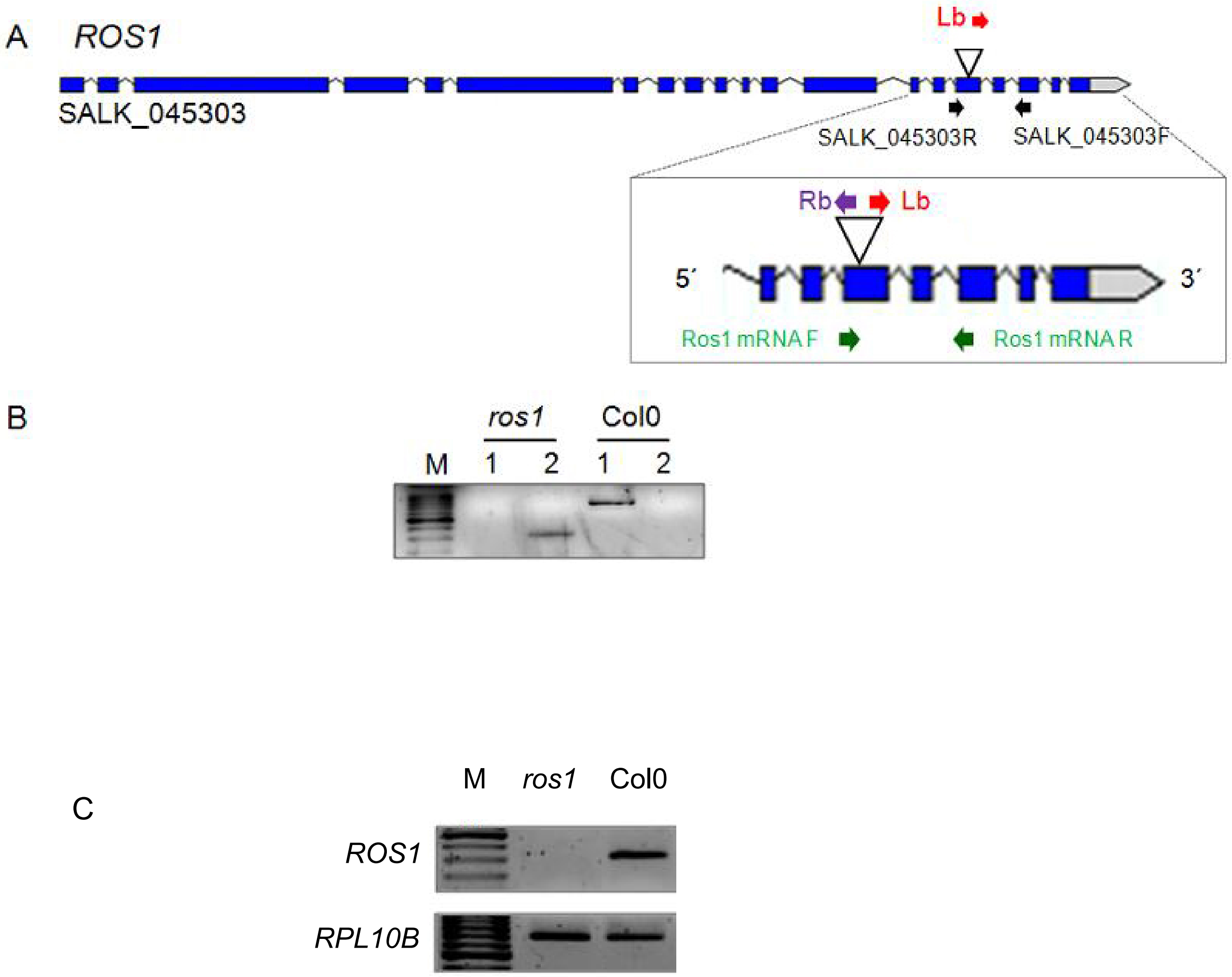

A
ROS1
Lb
SALK_045303
SALK_045303R
SALK_045303F
Rb
Lb
5´
3´
Ros1 mRNA F
Ros1 mRNA R
B
ros1
Col0
M
1
2
1
2
C
M
ros1
Col0
ROS1
RPL10B

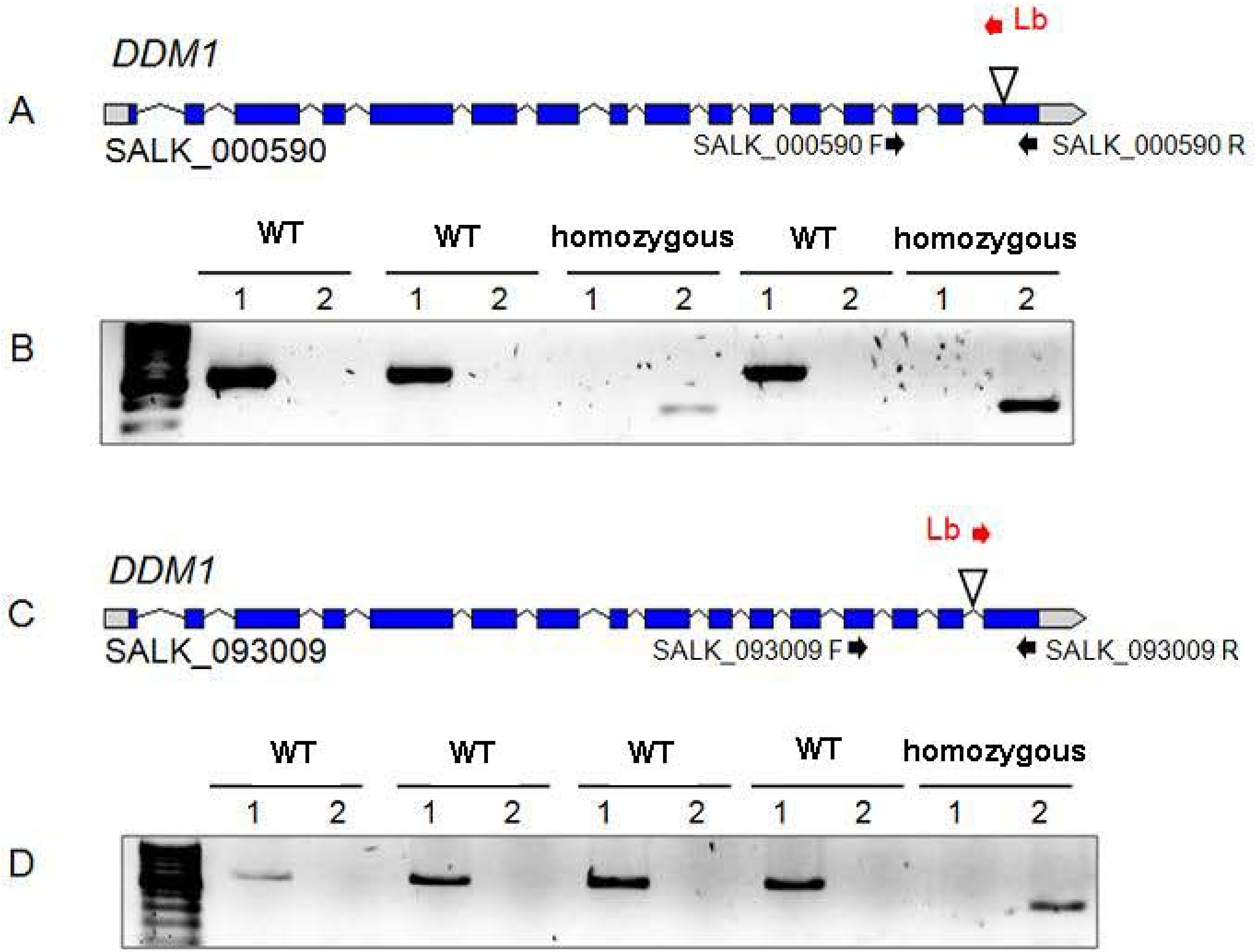
DDM1
A
Lb
SALK_000590
SALK_000590 F
SALK_000590 R
WT
WT
homozygous
WT
homozygous
1 2 1 2 1 2 1 2 1 2
B
DDM1
C
Lb
SALK_093009
SALK_093009 F
SALK_093009 R
WT
WT
WT
WT
homozygous
1 2 1 2 1 2 1 2 1 2
D

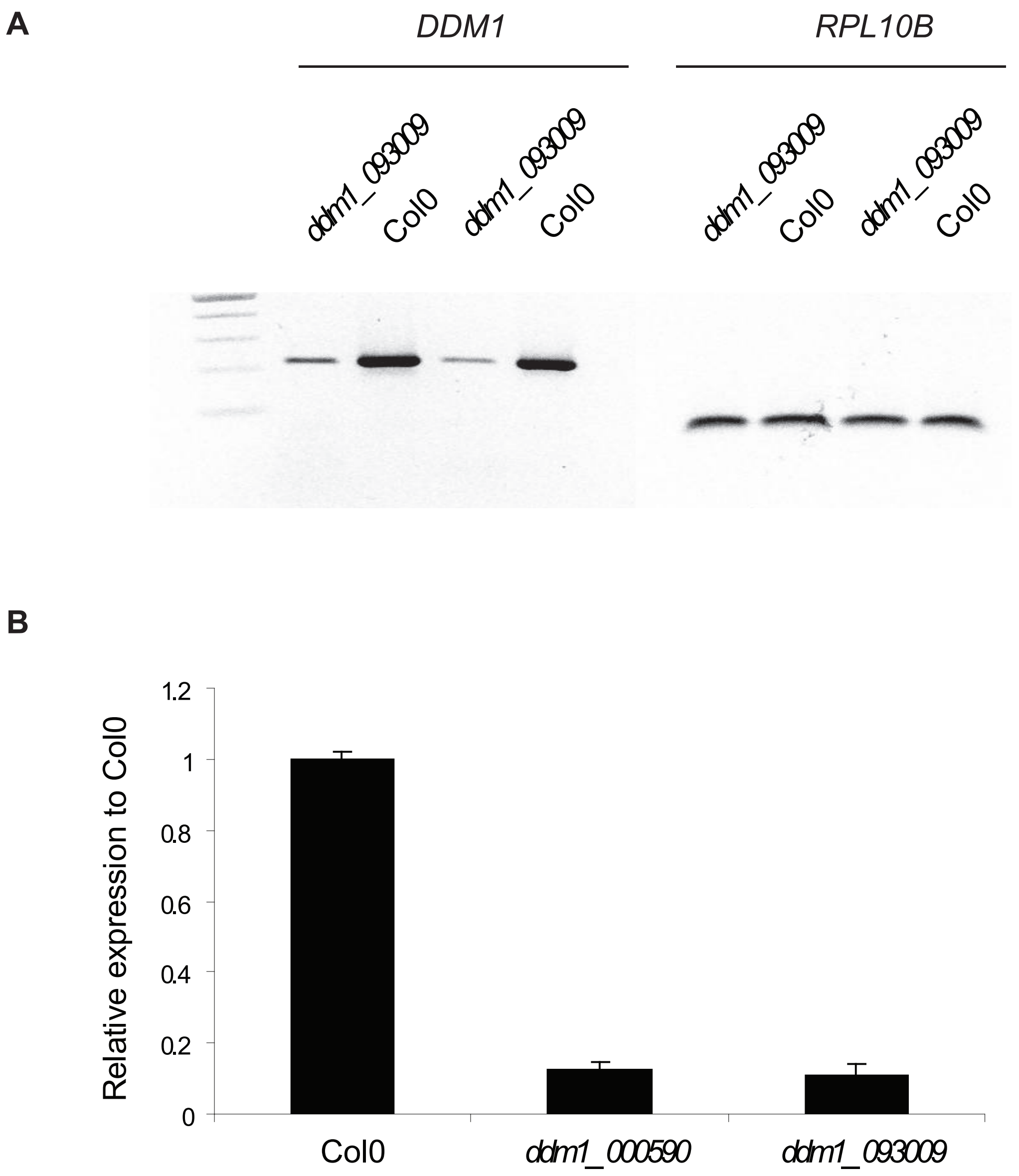
A
DDM1
RPL10B
ddm1_093009
Col0
ddm1_093009
Col0
ddm1_093009
Col0
ddm1_093009
Col0
B
Relative expression to Col0
1.2
1
0.8
0.6
0.4
0.2
0
Col0
ddm1_000590
ddm1_093009

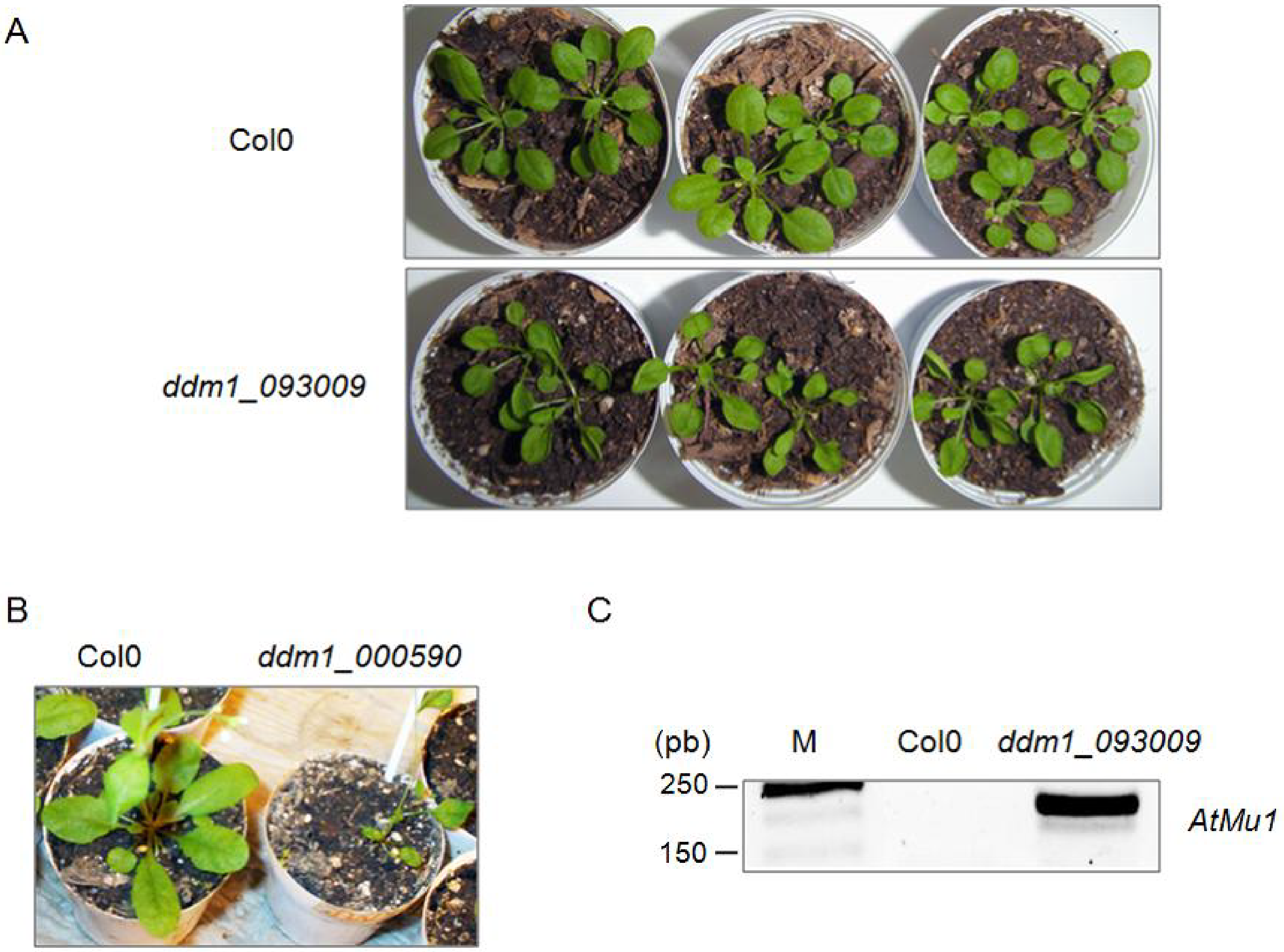
A
Col0
ddm1_093009
B
Col0
ddm1_000590
C
(pb)
M
Col0
ddm1_093009
250
150
AtMu1

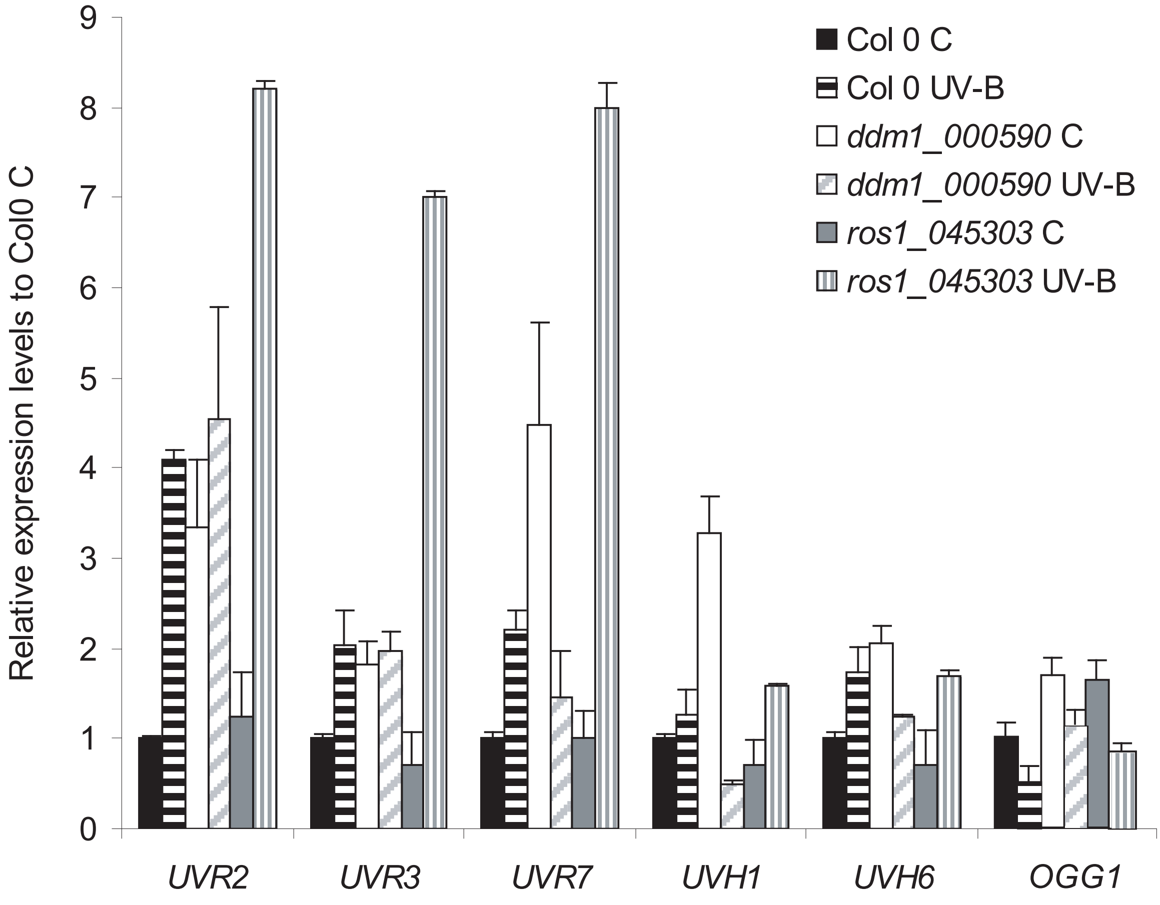

Col 0 C
Col 0 UV-B
ddm1_000590 C
ddm1_000590 UV-B
ros1_045303 C
ros1_045303 UV-B
Relative expression levels to Col0 C
0
1
2
3
4
5
6
7
8
9
UVR2
UVR3
UVR7
UVH1
UVH6
OGG1

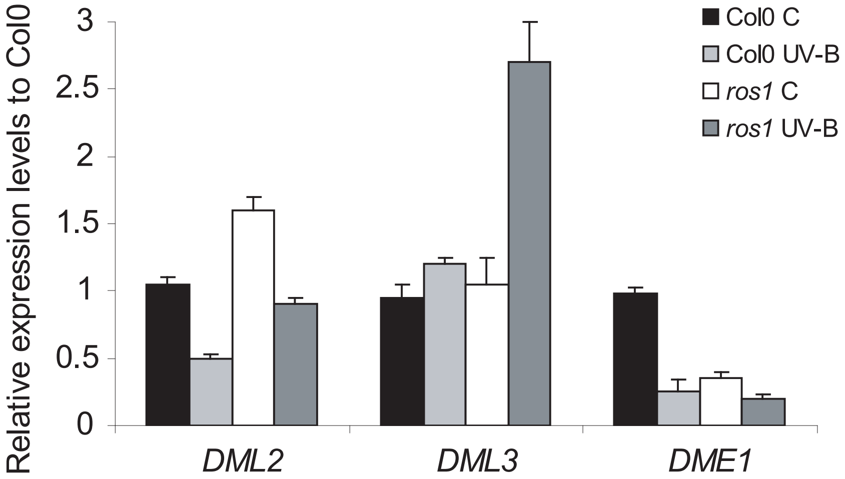

Relative expression levels to Col0 C
3
2.5
2
1.5
1
0.5
0
DML2
DML3
DME1
Col0 C
Col0 UV-B
ros1 C
ros1 UV-B

Table S1. Primers used for identification of homozygous mutant lines

Name	Sequence
LB SALK	GTCCGCAATGTGTTATTAAGTTGTC
SALK_045303 F	CATGATCCGCAAACACCT
SALK_045303 R	CAGGTGAGACGGCTGATT
SALK_135293 F	TTCTTTGGTCCGGTTGAA
SALK_135293 R	GGACCGAAGCCTTTGATT
SALK_093009 F	TGGAGGACTCGGAATCAA
SALK_093009 R	TCTCTCCCGGTGCAGTAA
SALK_000590 F	CCAGAACCCTCAAATGGAC
SALK_000590 R	CTCCCGGTGCAGTAATTG

Table S2. Primers used for RT-qPCR

Name	Sequence
ROS1 F	TCTCCTGCAACAGCATCA
ROS1 R	CATGATCCGCAAACACCT
DDM1 F	CAAGGCTGGAAGGGAAAG
DDM1 R	TCGTCCATTGGGAGAAGA
UVR2 F	GACCCGAGTGGATATGTTGG
UVR2 R	GAGCTGTTCTTCAGCTTTCC
UVR3 F	TTCAACCGCATCTACTCTCC
UVR3 R	TCTTCCTCTTGCACTCCTTG
UVR7 F	TACATTCGGGTCTCTTGCTC
UVR7 R	TCCTCGTCTTCTTCAACAGG
UVH1 F	CCAGAGCTTCACATCAGGTC
UVH1 R	ACCTTAACCGAGGAAAATGC
UVH6 F	CAAAGGCTGATTATGGGATG
UVH6 R	CACCAGTCTCAGCCATCTTC
DML2 F	ATTGAGGAACCAGCATCACC
DML2 R	GCTCTGTGCGTAGCTTTTCC
DML3 F	AGGGGCCTTTCCACTTAATG
DML3 R	GCTCTTTTGGATCTCGTTGC
OGG1 F	CTGAAAACCGACAAACCTGT
OGG1 R	AACAGCAAATCTCCACAAGG
RPL10B F	TGGTGTTCCCGATCCTAA
RPL10B R	ATCTTTCCCGGCAGACTT
CDPK3 F	CGCTGAGAACCTTTCTGAAG
CDPK3 R	CCATCTCCATCCATATCAGC
AtMu1 F	CAAGACCTGTGGTGAAGCTG
AtMu1 R	CTTGAGAAGGTTGTGTGATG
